# Empowerment and social inclusion through Para sports: a qualitative study on women with physical impairments in Saudi Arabia

**DOI:** 10.3389/fpsyg.2024.1366694

**Published:** 2024-05-02

**Authors:** Majed M. Alhumaid, Yuhanis Adnan, Mohamed A. Said, Maryam A. Alobaid, Selina Khoo

**Affiliations:** ^1^Department of Physical Education, College of Education, King Faisal University, Al-Ahsa, Saudi Arabia; ^2^Faculty of Sports and Exercise Science, Universiti Malaya, Kuala Lumpur, Malaysia

**Keywords:** social inclusion, women Para-athletes, disability, physical disability, Saudi women

## Abstract

**Background:**

Participation in sports represents a potent means of empowerment and social inclusion. Nevertheless, women with physical impairments encounter specific challenges in accessing Para sports. The main aim of this study is to present the experiential participation and achievements in sports of women with physical impairments in Saudi Arabia.

**Methods:**

Twenty women athletes with physical impairments who engaged in competitive Para sports in Saudi Arabia were interviewed. Interpretive phenomenological analysis was employed to extract themes elucidating the experiences of women athletes with physical impairments in Para sports.

**Results:**

Four dimensions were identified: (i) Exploring participation in sports; (ii) The positive impact of participation in sports; (iii) obstacles in participation in sport; and (iv) hopes and aspirations to improve participation in Para sports.

**Conclusion:**

In Saudi Arabia, participation in Para sports functions as a powerful tool for empowering and socially integrating women with physical impairments. However, these women encounter challenges in accessing sports. Achieving empowerment in Para sports necessitates the establishment of an inclusive ecosystem that celebrates diversity and equality. Collaborative efforts from governments, sports organizations, communities, and individuals are indispensable in creating an environment where women with impairments can flourish in sports.

## 1 Introduction

The World Health Organisation defines disability as an umbrella term for impairments, activity limitations, and participation restrictions that indicate the negative aspects of the interaction between an individual (with a health condition) and that individual’s contextual factors (environmental and personal factors) (World Health Organization, 2011). It is estimated that there are 1.3 billion people in the world who experience a significant disability (World Health Organization, 2023, March 7). Notably, the prevalence of disability tends to be higher among women than men, with the average prevalence of women aged 15 years and older is 18%, compared to 14.2% for males (World Health Organization, 2022). This means that about 1 in 5 women have a disability. Women with disabilities are a diverse group with a wide range of experiences, abilities, and needs, as well as unique challenges and opportunities. However, it is apparent that women with disabilities experience greater challenges and have considerably poorer experiences than their male contemporaries, facing greater barriers to access and participation (Emmett and Alant, 2006). Wickenden (2023) described disability, like other identities, as socially constructed, subject to misrepresentation, and frequently isolated from other issues and experiences.

Sports are a social activity that provides a platform for societal activity engagement, allowing for a sense of individual and collective self, meanings, and participation through social interaction (Svanelöv et al., 2020). Para sport refers to the athletic activities that are engaged in by individuals who have a qualifying physical, visual, or intellectual impairment, as governed by the International Paralympic Committee and its member organizations (Vanlandewijck and Thompson, 2011). Active participation in sports plays a vital role for people with disabilities in shaping their athletic sense of self, improving both physical and mental well-being, and significantly enhancing the overall quality of life (Ruddell and Shinew, 2006; Martin, 2013; Diaz et al., 2019; Martin Ginis et al., 2021). Para sport is a dynamic and inclusive arena within the athletic landscape that celebrates the athletic abilities and achievements of people with disabilities (International Paralympic Committee, 2023). It gives athletes with disabilities a stage to demonstrate their ability, determination, and tenacity, inspiring others and breaking down preconceptions while promoting equality and inclusion. Para sports not only redefine athleticism but also develops a sense of empowerment and camaraderie among participants and spectators. There are local and various international competitions for athletes with disabilities, including multi-sport and multi-disability competitions (e.g., Paralympic Games) and single disability competitions (e.g., Deaflympics and Special Olympics World Games). Along with competing in multi-sport events, disabled athletes also take part in a plethora of world cups and championships, including but not limited to the UCI Para-Cycling Track World Championship, the INAS World Football Championship, the IPC Powerlifting World Championship, the World Para Table Tennis Championship, and so on.

Although women athletes with disabilities have made great advances in recent years, they still face challenges such as unequal media coverage, limited participation opportunities, issues of intersectionality, insufficient financing, and a need for increased activism (DePauw, 2000; Roy, 2011; Moodley and Graham, 2015; Kirokosyan, 2021; Weiller-Abels et al., 2021; Alhumaid et al., 2022). To properly empower and celebrate women athletes with disabilities, it is critical to address these concerns and work toward a more inclusive and equitable sports scene where their accomplishments are recognized and celebrated in the same way as those of all other athletes. Empowerment, as defined by Kosciulek and Merz (2001), is the result of a combination of internal factors such as a sense of control, competence, responsibility, commitment, and future orientation, as well as situational factors that encompass social elements like control over resources, interpersonal skills, work, organizational skills, and social skills. These qualities contribute to enhanced community inclusion, empowerment, and overall well-being for individuals with disabilities (Kosciulek and Merz, 2001).

However, the participation of women with disabilities in sports is complex, and affected by the intersectionality of gender and disability. It is important to consider not only the specific nature and severity of the disability but also the specific sports they pursue (Emmett and Alant, 2006; Kirokosyan, 2021; Ballas et al., 2022; Olasagasti-Ibargoien et al., 2023; Richard et al., 2023). Kirokosyan (2021) noted that fewer women than men participate in Para sports, and the causes for this disparity are compounded by various factors, including gendered power dynamics, societal and cultural barriers, belief systems, and women’s personal decisions. These variables, alone or in combination, may influence the women’s participation in sports and potentially hamper efforts to raise awareness of the issues distinctive to female athletes with disabilities (Seal, 2012; Moodley and Graham, 2015; Kirokosyan, 2021). DePauw (2000) observed that despite being a smaller group compared to male athletes with disabilities, women have actively engaged in Para sports since the early 1920s. However, women athletes with disabilities aiming for high-level sports competition often confront dual discrimination rooted in both their gender and disability. More than three decades ago, Wendell (1989) remarked that women with disabilities face dual challenges: they grapple with the subjugation associated with being women in predominantly male-dominated societies and also contend with the oppression stemming from their disabilities in societies largely dominated by individuals without disabilities.

In Saudi Arabia and other arab countries, women athletes with disabilities face a unique set of challenges and barriers in the realm of sports. According to Alhumaid et al. (2022), the challenges faced by female athletes with disabilities in Saudi Arabia can be exacerbated by cultural, religious, and gender-specific issues as well as a lack of awareness and representation. Traditional gender roles and societal attitudes toward women in Saudi Arabia can create barriers for women athletes with disabilities. Stereotypes and biases may discourage their participation in sports and limit their access to training and competitive opportunities (Mohamed et al., 2020; Zahra et al., 2022). Some families and communities may discourage or even prohibit women, particularly those with disabilities, from engaging in sports. As a result, female athletes with disabilities generally find themselves little known or visible in Saudi Arabia. This lack of representation can contribute to the marginalization of these athletes and hinder their recognition and support from the wider society.

Nevertheless, the research on the quality of involvement of women with disabilities in the Arab region remains reduced in both quantity and quality. Further investigation is required to fully comprehend the extent of involvement among these women in society, taking into consideration ethnic diversity, cultural factors, and geographical location (Gharaibeh and Remaih, 2022). It is also unclear how the impact of the interplay between individuals and their surroundings on participation is essential for fostering full engagement in Para sports. Additionally, there is a research gap regarding gender equity in sports specific to Saudi Arabia, the broader Middle East, and other countries of the Arabic region (Alruwaili, 2023).

The main aim of this study is to present the experiential participation and achievements in sports of women with physical impairments in Saudi Arabia as physical impairment is the most common disability in the country (Unified National Platform, 2023). The current study delves into their participation in sports, both recreationally and competitively, exploring the benefits and challenges they face. It also discusses the influence of traditions, cultural norms, and expectations on women with physical impairments in the context of sports. Furthermore, it addresses the future development of Para sports and the processes that have impacted the inspiration and empowerment of women with physical impairments. This study represents a new transformative, focusing on recent advancements in sports for women with physical impairments in Saudi Arabia.

## 2 Materials and methods

### 2.1 Procedure

After receiving ethical approval from the Research Ethics Committee at King Faisal University (KFU-REC-2023-JUN-ETHICS1091), women with physical impairments who were actively engaged in sports in Saudi Arabia were invited to take part in this qualitative research project. Participants were recruited through sports associations for persons with physical impairments in Saudi Arabia. Those who were willing to participate were sent an information sheet detailing the study’s objectives and purpose, along with an informed consent form for them to sign. As the participants resided in different cities across Saudi Arabia, all interviews were conducted via telephone and no incentives were given for participation. The interviews, typically ranged from 25 to 30 min in duration, were audio-recorded with permission from participants and subsequently transcribed verbatim.

### 2.2 Participants

Saudi Open Data Portal (2023) reported that there were 53 Paras ports clubs associated with the Saudi Arabian Paralympic Committee, as stated by the Ministry of Sports. These clubs focus on 23 specific parasports and include a total of 3,682 athletes, with the majority being male. Out of all the clubs mentioned, only 7 of them have women’s teams that participate in 4 distinct parasports, resulting in a combined total of 54 athletes. The sports comprised of deaf bowling with a participation of 2 athletes, 4 track and field clubs with a total of 45 participants, weightlifting with 4 athletes, and table tennis with 3 players.

A total of 34 athletes were contacted by our research team, and out of these, 20 agreed to be interviewed. Potential participants were required to satisfy the following inclusion criteria: (a) be at least 18 years old, (b) be currently engaged in sports, (c) have a physical impairment. The participants were between the ages of 19 and 50 (*M* = 36.3 years; *SD* = 8.62 years). All participants reported that they receive attendant care; when asked to self-rate their health status, more than half (65%) described their health as ‘Good’; the remaining participants reported being in ‘Excellent’ health. Most of the participants (75%) had an acquired disability, while others had had a disability since birth. Demographic information of participants is shown in Table 1.

**Table 1:** Demographic profile of participants.

Variable	Categories	Number (%)
Age	19–30 years old	5 (25)
	31–40 years old	7 (35)
	More than 40 years old	8 (40)
Education level	Middle school	1 (5)
	High school	7 (35)
	Diploma	2 (10)
	Bachelor	10 (50)
Occupation	Unemployed	7 (35)
	Student	2 (10)
	Athlete	1 (5)
	Self-employed	1 (5)
	Government employee	3 (15)
	Private sector employee	6 (30)
Marital status	Single	11 (55)
	Married	5 (25)
	Divorced	4 (20)
Years living with disability	<5	4 (20)
	5–10	1 (5)
	11- < age	9 (45)
	Since childhood	6 (30)
Sport participation level	District	4 (20)
	Club	10 (50)
	International	6 (30)
Years of playing sports	1–2	13 (65)
	3–4	1 (5)
	5–7	5 (25)
	8–9	1 (5)
Type of sports	Athletics	14 (70)
	Table Tennis	3 (15)
	Bowling	1 (5)
	Weightlifting	2 (10)
Self-rated physical activity	Moderately active	6 (30)
	Active	12 (60)
	Highly active	2 (10)
Time of play sports (days/week)	3	5 (25)
	4	4 (20)
	5	5 (25)
	6	6 (30)
Time of play sports (hours/day)	2	9 (45)
	3	6 (30)
	4	4 (20)
	5	1 (5)
Achievements	None	9 (45)
	Medal(s) in local championships	7 (35)
	Medal(s) in the national championship	2 (10)
	Medal(s) in the international championship	2 (10)

### 2.3 Measures

In order to investigate the participants’ experiences, viewpoints, and attitudes toward participation in sports in Saudi Arabia, comprehensive individual interviews using a guided approach were carried out. This involves basing the interview topics on the interviewees’ respective backgrounds to enhance the thoroughness and precision of multiple interviews (Patton, 1990). Following McGrath et al.’s (2019) recommendation, the interviews began with relatively straightforward warm-up questions to establish rapport between the interviewer and the interviewees. Subsequently, the questions then moved to more specific topics to elicit the participants’ experiences, perspectives, and views on sports in Saudi Arabia.

The interview questions covered various topics, including the psychological, physical, and social impacts of sports participation, as well as the challenges and barriers encountered by the participants in relation to their involvement in sports. Below are some examples of the interview questions:

1. What do you think of the sports program that you participate in?
2. What do you like most about playing the sport?
3. Do you feel any changes (physical, mental) in yourself after playing the sport? If yes, can you describe these changes?
4. Do you face any issues related to your participation in sports?
5. What do you think would encourage more women with physical impairments to participate in sports?

The interviews were concluded once data saturation was achieved, indicating that no new themes or significant information were emerging from the interviews. However, to ensure thoroughness and confirm the absence of any overlooked themes, three additional interviews were conducted. These supplementary interviews served as a precautionary measure to validate the completeness of the data and ensure that all possible themes had been adequately explored and captured (Jassim and Whitford, 2014).

### 2.4 Data analysis

To explore the experiences, perspectives, and opinions of women with physical impairments engaged in sports in Saudi Arabia, an interpretive phenomenological analysis approach was utilized (Smith et al., 2009). Rather than simply aiming to confirm or reject a specific hypothesis, interpretative phenomenological analysis entails researchers endeavoring to organically capture and explore the meanings that participants ascribe to their encounters with the subject of investigation (Reid et al., 2005). In this particular case, the focus was on the experiences of women with physical impairments participating in sports in Saudi Arabia. These meanings were subsequently categorized into distinct themes to offer a comprehensive overview of the subject under investigation (Braun et al., 2016).

In the present study, we used a five-step process to identify and categorize the principal themes. First, we thoroughly read and reread the interview transcripts to become familiar with the content. Second, we sought to identify recurring patterns, both common and less common, that yielded valuable insights into the participants’ viewpoints, perspectives, and experiences related to participating in sports in Saudi Arabia. Each distinct theme was assigned a unique code. Third, we inductively determined and organized these emerging codes (Saldaña, 2013) to formulate preliminary themes. Fourth, these preliminary themes were clarified and labeled. Finally, in the fifth step, the preliminary themes were organized into the overall themes (dimensions).

### 2.5 Trustworthiness

Trustworthiness of the findings was ensured using two methods: member checks and peer review. First, member checks were employed to enhance the credibility of the findings. The interview transcripts were returned to all participants to validate, rectify, deny, or elaborate on the information they provided during their interviews. Second, peer review or debriefing, as an external assessment of the data (Lincoln and Guba, 1985; Creswell, 2007), involved an experienced qualitative researcher who is an associate professor in the field of teaching methods at a Saudi university. With 10 years of experiences in qualitative research, the peer reviewer evaluated the integrity of the study. The peer reviewer also collaborated with the coders during the five-step process to identify and categorize the main themes.

### 2.6 Reflexivity

Reflexivity plays a pivotal role in qualitative research, serving as a mechanism for researchers to critically examine their own assumptions, biases, and beliefs that could influence the research process and outcomes (Creswell and Miller, 2000). In this study, reflexivity was integrated into the research design to inform both data collection and analysis (Tracy, 2010). To minimize potential biases, the authors conducted a bracketing interview, creating a structured platform for introspection and reflection on theoretical assumptions and biases. Following this initial step, collaborative team discussions were conducted to delve deeper into these identified biases and assumptions, ensuring a thorough exploration and effective mitigation of their potential impact on the study’s findings. Furthermore, the research team for this study comprises individuals with physical/mobility disabilities, who are also former Paralympian and research sport psychologist, alongside academic experts in the fields of adapted physical activity and sport, as well as sport sociology, particularly in the area of disability sport. This diverse composition brings a unique perspective and depth to the research process, enriching the study’s overall rigor and validity.

## 3 Results

The interpretative phenomenological thematic analysis (Reid et al., 2005) revealed four main dimensions that the 20 participants associated with their experiences as women with physical impairments participating in sports in Saudi Arabia. These dimensions were extracted from the 50 most frequently occurring themes and sub-themes drawn from the raw data; these were organized into 12 first-order sub-themes and 9 s-order sub-themes These first- and second-order themes are presented and exemplified by representative interview extracts (see Table 2). The four main dimensions are as follows: (i) *Exploring participation in sports*, (ii) *The positive impact of participation in sports*, (iii) *The challenges of participating in sports*, and (iv) *Motivations to participate in sports*.

**Table 2:** Participants’ experiences of participating in sports.

Raw data themes	1st order subthemes	2nd order subthemes	Dimensions
Introduced by friends and colleagues (*n* = 9) Explored by social media (*n* = 4) Introduced by associations and organizations for people with disabilities (*n* = 5) Introduced to clubs for people with disabilities (*n* = 5)	Introduction to sports	Exploring participation in sports	Exploring participation in sports
It is a great feelings (*n* = 9) Feelings toward sports changed from negative to positive (*n* = 7) Improved mental health (*n* = 3) Psychological comfort, positive energy, and getting rid of stress and negative energy (*n* = 17) Improvement in concentration, reflexes, and responsiveness (*n* = 6) My outlook toward myself and people with disabilities has become more positive (*n* = 2) I feel like I have a purpose in life (*n* = 1) For a more useful and enjoyable life (*n* = 9)	Psychological effects of sports participation	Health benefits	The positive impact of participation in sports
Improving physical health (*n* = 3) Losing weight (*n* = 6) Increasing physical activity, strengthened muscles, physical fitness, healthy body (*n* = 12) Strengthening muscles through movement, activity, vitality, health and fitness (*n* = 6) Moving with the wheelchair and moving has become faster and easier than before (*n* = 11) Improvement in balance (*n* = 6) My body has become stronger and more flexible (*n* = 17)	Enhanced physical health and fitness		
Reduced pain and dispensing with medical treatments (*n* = 3)	Medical impact		
Time management (*n* = 15) Family support (*n* = 3) Paying attention to nutrition (*n* = 1) Intensive camps and ongoing coaching support (*n* = 3) The coaches are at a very high level and efficient (*n* = 18) Availability of devices and equipment (*n* = 7) Like the exercises and training program (*n* = 14) Love of sports (*n* = 2) The program supports and encourages women with disabilities to participate in sports (*n* = 4) Excellent, enjoyable, stimulating (*n* = 17)	Key factors that contribute to sports participation	Key factors that contribute to sports participation	
Building friendship and social relationships (*n* = 2) Like the social aspect and exchange of experiences with others (*n* = 14)	Enhanced social connections	Social benefits	
Encourage friends and others to do sports and join the sports clubs (*n* = 15) Invite women with disabilities to participate in the experience (*n* = 5)	Promoting Para sports		
Exercising and training result in increased physical effort and psychological stress (*n* = 4)	Negative effects of training	Health impacts	Challenges in participation in sports
Transportation issues (*n* = 8) Financial issues (*n* = 3) Difficulty obtaining assistive devices (*n* = 1)	Logistical challenges	Logistical challenges	
Changing society’s perception and attitudes toward disability and people with disabilities (*n* = 1) Improving social awareness in society of people with disabilities and their rights (*n* = 3)	Promoting awareness and changing societal perceptions of disabilities	Social awareness	Motivation to participation in sports
Determination and persistence to achieve goals (*n* = 2) To make sporting achievements (*n* = 6) To represent the country in international Paralympic competitions (*n* = 12) Become a world-class Paralympian (*n* = 11) To become an assistant coach (*n* = 1) Continuing sports and striving to make achievements (*n* = 5) To be an active, an effective member, and a role model in society (*n* = 2)	Personal motivation – intrinsic and extrinsic	Personal motivation – intrinsic and extrinsic	
Paying more attention to sports clubs for people with disabilities (*n* = 3) Providing social and psychological specialists (*n* = 1) Empowering Saudi female coaches (*n* = 2)	Future development	Future development	

### 3.1 Exploring participation in sports

This dimension provided evidence about how the participants had become involved in sports. Specifically, several participants reported that they had initially been introduced to sports through colleagues and friends. As one participant mentioned, *‘… it was by my friend. I wasn’t aware that there was a club for people with disabilities that offered sports and exercises. However, my friend informed me of its existence and recommended that I go, so I did.’* Social media also provided a useful way for participating to discover opportunities to participate in sports. One participant mentioned, ‘… it was by chance, while browsing a social networking site, I came across video clips of Saudi girls with physical impairments participating in a local tournament. This ignited my desire to join in.’ In addition, various sports clubs, associations, and organizations for those with disabilities emerged as a factor in attracting the participants to become involved in sports. As one participant stated:

*‘There was an event at the club for people with disabilities, and during the event, a coach approached me and invited me to participate in a game. After being tested, I signed a contract with XX club, and now I am part of the national team.’*

### 3.2 The positive impact of participation in sports

The positive impact of participation in sports, rooted in theoretical perspectives such as social capital theory and psychological well-being frameworks, is a central theme that emerged from the analysis of 30 raw data themes. Before delving into the description of each second-order sub-theme, it is essential to provide a comprehensive understanding of the overarching concepts that underpin this theme. In this section, we will explore the various *health benefits*, *key factors that contribute to sport participation*, and *social benefits* associated with engaging in sports activities. By examining these dimensions, we aim to shed light on the multifaceted positive impact that sports participation can have on women with physical impairments.

#### 3.2.1 Health benefits

The health benefits of participation in sports emerged as a key theme across various health dimensions, including psychological, physical, and medical. To illustrate this, numerous participants highlighted the positive psychological impacts of participating in sports including feelings of psychological comfort, positive energy, alleviating stress and negative emotions, and providing a purpose in life. Specifically, one participant mentioned, ‘I feel a sense of happiness during and after playing group games with individuals who share the same disability and level of thinking, which generates positive energy and overall happiness.’ Additionally, another participant expressed that:

*‘Undoubtedly, my life has undergone significant changes after engaging in sports, and my perspective on life has evolved. Previously, I held the belief that individuals with disabilities lacked purpose, but after experiencing sports, my viewpoint completely transformed. Now, I recognize that individuals with disabilities can pursue meaningful careers and achieve their goals.’*

Meanwhile, several participants mentioned the noticeable improvement in their physical well-being. For instance, one participant asserted that she has become swifter and lighter in her movements and that her mobility has improved because of her participation in sports. Besides, another participant expressed, *‘Participating in sports has brought substantial changes to my life. My physical fitness has improved, making it easier for me to maneuver my wheelchair with greater agility and speed.’* Reduced pain and the need for medical treatments were also cited as significant benefits of participating in sports, as one participant asserted, *‘Not only has my pain subsided, but I have also been able to avoid surgeries related to my scoliosis and reduce the number of medical treatments I require due to my engagement in sports.’*

#### 3.2.2 Key factors that contribute to sport participation

Several factors significantly contributed to the participants’ involvement in sports and had positive impacts on their overall experiences. One of the key factors was the development of effective time management skills. Participants emphasized that effective time management and organization of daily tasks played a vital role in enabling their continued sports participation. As an illustration, one participant noted, *‘I would ensure that I completed all my household duties early in the day, allowing me more time to go to the club.’* This highlights how efficient time management facilitated their commitment to sports and created opportunities for dedicated practice and engagement.

Moreover, participants highlighted the importance of coaches’ professionalism and consistent dedication to athlete development and performance enhancement. The unwavering support and guidance these coaches provided significantly enhanced the participants’ participation in sports. Participants noted that their coaches were understanding, cooperative, and patient, going above and beyond to help each athlete improve. One participant reported, *‘Our coach is incredibly supportive, even when unexpected circumstances arise. He is understanding, cooperative, and patient, often repeating exercises to help each athlete improve.’* Relatedly, another participant expressed that:

*‘The coaching staff displays exceptional character. One coach has been with me since 2018, and he is incredibly motivating. Before each training session, he thoroughly assesses the athlete’s needs, considering their physical condition and specific disabilities. This personalized approach by the coach greatly boosts our enthusiasm for training, as they tailor exercises to suit both our health condition and disability type.’*

In addition to effective time management and supportive coaches, strong and continuing family support emerged as a crucial factor in encouraging participation in sports. Participants expressed gratitude for their families’ genuine passion for sports and training, which played a pivotal role in their ongoing sports engagement. One participant emphasized:

*'My entire family shares a genuine passion for sports and training. Their unwavering support has been instrumental in my ongoing sports participation. I have even introduced my daughter to the world of sports, and my son is the reigning champion in Romanian wrestling in our country. Our household is a sports-oriented family that values athletic achievements.'*

This illustrates how family support creates an environment that fosters and nurtures sports participation, contributing to the participants’ positive experiences and achievements. To end, these factors, including effective time management skills, coaches’ professionalism and dedication, and strong family support, play a critical role in enhancing participants’ sports participation. They contribute to positive outcomes such as personal growth, skill development, and enjoyment. Understanding and addressing these key factors are essential for comprehending the overall positive impact of sports engagement. By acknowledging and incorporating these factors into sports programs and interventions, it is possible to create an environment that maximizes the benefits and positive experiences derived from sports participation.

#### 3.2.3 Social benefits

The findings also revealed that participating in sports provides numerous social benefits for individuals with disabilities. Such benefits include building relationships, improving communication skills, increasing awareness, and fostering a sense of belonging. The participants mentioned several social benefits that resulted from participating in sports; these included enhancing social connections and promoting the role of sports for individuals with disabilities within the community. For instance, one participant shared:

*'It [participation in sports] has also had a significant impact on the social aspects of my life. It has allowed me to connect with individuals who have disabilities, enabling us to exchange experiences within the club. This exchange occurs not only among individuals with similar disabilities but also among those with different disabilities. It has allowed us to share our experiences and strategies for overcoming challenges.'*

Another participant emphasized the social benefits she experienced:

*'The social aspect should not be underestimated, as it has a profoundly positive psychological impact on us as individuals with disabilities. We were once confined to our homes, with limited interactions within our families. However, now we have cultivated relationships and acquaintances that extend beyond our immediate family and relatives.'*

Moreover, the participants unanimously agreed that participating in sports is crucial for fostering better interpersonal relationships and friendships. For example, one participant asserted that participating in sports had significantly improved her social relationships and friendships as she had become more confident in asking her peers about unfamiliar sports-related matters and seeking guidance from more experienced peers––especially as she is new to the club––as well as providing opportunities to meet others who share the same disability. In addition, the participants strongly emphasized promoting involvement in sports for women with disabilities, particularly those with physical impairments. For example, one participant, who works at a Saudi university, stated:

*'Since I began my involvement in sports, I've been actively working to invite, encourage, and engage women with disabilities to participate in sports activities at our club. I've organized various events and friendly tournaments within the University, along with awareness campaigns to highlight the availability of sports for individuals with disabilities, regardless of the severity of their condition. These efforts have successfully encouraged numerous women to join and engage in sports. I take great pride in these endeavors and remain committed to this cause.'*

### 3.3 Challenges in participation in sports

The findings in this dimension highlighted the participants’ experiences related to the challenges they face due to their participation in sports, including *health impacts* and *logistical challenges*.

#### 3.3.1 Health impacts

The first challenge mentioned by the participants related to the physical and psychological impacts associated with rigorous physical training and involvement in sports. For instance, as one participant, a shot-put athlete, mentioned, *‘…some exercises require intense physical effort, which can create psychological pressure…, I understand that these exercises are for the benefit of the athlete, but they can be mentally challenging.’* Another shot-put athlete mentioned that she experienced negative impact because of the high-intensity training in internal and external local and international sports camps.

#### 3.3.2 Logistical challenges

The second challenge mentioned by several participants was the logistical difficulties they encountered when participating in sports, which sometimes affected their performance. For instance, one participant mentioned the transportation-related difficulties when going to her sports club, highlighting that the effort involved in arriving on time often leads to psychological pressure. To address this issue, another participant suggested, *‘We need to reconsider transportation because our wheelchairs require a hoist to be loaded into a vehicle. Therefore, dedicated transportation solutions are necessary.’*

Participants also raised concerns about financial sufficiency and the lack of provision of assistive devices for sports participants with disabilities. In particular, they asserted that greater attention and support are needed from relevant organizations to enable clubs to support athletes financially to enable them to feel more secure about attending training and competitions and potentially attract more individuals with disabilities to participate in sports. Access is a frequently mentioned obstacle in para sport and is connected to several factors (Jaarsma et al., 2014; Conchar et al., 2016; Diaz et al., 2019). Initially, there is a substantial financial investment required to engage in adaptive sports. The cost of adaptive equipment is high, and it is typically customized for everyone, which makes it challenging to share among numerous participants. Regular gyms frequently lack access to this equipment, or they are not designed to cater to certain requirements due to disarray and other inadvertent circumstances. While the prevalence of adaptive sports leagues is increasing, they are not universally available and may necessitate substantial travel time for participation.

The limited availability of opportunities is particularly pronounced for female participants, as most adapted sports leagues are predominantly male dominated (Yoh et al., 2008). Transportation options may be restricted for individuals in this demographic due to their inability to operate vehicles, necessitating dependence on others or public transportation (Martin Ginis et al., 2012). Jenkins (2002) affirmed that the significance of sporting equipment extends much beyond its basic functionality. It is a crucial component of an athlete’s performance, ensuring their safety and adherence to sport-specific regulations. Nevertheless, the economic significance of sports should not be undervalued, especially in terms of the equipment requirements of individuals aspiring to achieve the remarkable accomplishments of Paralympic competitors. Racing wheelchairs and other sports-specific mobility technology are costly and are generally not covered by health insurance. A significant number of individuals with disabilities are required to combine their financial resources to cover the expenses associated with their daily healthcare needs. Moreover, the exorbitant costs of adaptive sports technology serve as a barrier, restricting the number of individuals who may pursue their aspirations as athletes.

### 3.4 Motivation to participate in sports

Motivation to participate in sports, rooted in theoretical perspectives such as self-determination theory or achievement goal theory, provides valuable insights into the factors and processes that drive individuals to engage in sports activities. It serves as a central theme that emerged from the analysis of 12 raw data themes and their respective 3 s-order sub-themes, namely *social awareness*, *personal motivation (intrinsic and extrinsic)*, and *future development*. Motivation, within the context of sports participation, refers to the intricate psychological processes and factors that initiate, guide, and sustain individuals’ engagement in sports activities. By understanding motivation, researchers and practitioners gain a crucial understanding of why individuals choose to participate in sports and what factors contribute to their continued involvement.

The three sub-themes further enrich the understanding of motivation in sports. Social awareness encompasses the influence of social interactions, norms, and support systems on an individual’s motivation to participate in sports. Personal motivation, comprising intrinsic and extrinsic aspects, delves into the internal desires and external incentives that drive individuals’ engagement in sports. Future development explores the aspirations, goals, and personal growth that individuals seek through their sports participation.

By comprehensively examining these sub-themes and their interrelationships, the conceptual framework provides a holistic understanding of motivation in sports participation. It elucidates how social factors, personal motivations, and future aspirations collectively shape individuals’ engagement and commitment to sports activities. This framework is a valuable tool for researchers and practitioners to analyze and interpret motivational processes, design targeted interventions, and optimize sports experiences for individuals involved in sports.

#### 3.4.1 Social awareness

Some participants expressed that their key motivations for participating in sports were to change the social perceptions surrounding people with disabilities being involved in sports and raise awareness about people with disabilities and their rights. The participants emphasized that increasing social awareness about the rights of individuals with disabilities was a crucial motivation for becoming involved in sports. For instance, one participant expressed her desire to contribute to raising social awareness and improving policies related to people with disabilities in sports and their rights. Another participant elaborated:

*‘…I aim to alter society’s perception of individuals with disabilities. In the past, there was a prevailing belief that individuals with disabilities were incapable of achieving anything – a misconception I personally experienced both in school and elsewhere. Through my involvement in sports, I aim to challenge this perception and demonstrate that disability is not a limitation of the body, but a misconception held by society… As a result of my efforts, I became the first Saudi woman to qualify for the 2020 Tokyo Paralympic Games, achieving a global sixth-place ranking…’.*

According to participants, the following factors increase athletes’ chances of successfully resolving social problems: (1) trust in athletes, (2) trust in supporting organizations, (3) awareness and willingness to respond to problem development, and (4) credibility and admiration for athletes as role models in their field. These characteristics are common among these athletes, who can deal with the challenges their country faces while maintaining high standards. Saudi athletes are very sensitive to social issues due to their daily encounters with disability-related challenges and barriers, their extensive knowledge of regional issues, and their potential to effect change in their country beyond the promotion of sport.

#### 3.4.2 Personal motivation – intrinsic and extrinsic

Various intrinsic and extrinsic motivations played significant roles in inspiring the participants to become involved in sports. One of the principal motivations was the participants’ aspirations to represent Saudi Arabia on the global stage at international Paralympic competitions. For instance, as one participant emphasized, *‘… my most cherished goal is to elevate Saudi Arabia’s reputation on the international stage through winning championships. I’m dedicated to achieving this goal with unwavering determination.’* Several participants cited their driving motivation for participating in sports in terms of their desire to become active and exemplary members of society. For instance, as one table tennis athlete articulated, *‘I embarked on my sports journey with the motivation to contribute to society as an active member and serve as a positive role model for others.’*

#### 3.4.3 Future development

During the interviews, several participants provided suggestions and recommendations to enhance and promote sports participation among individuals with disabilities in Saudi Arabia, especially women. For instance, one participant emphasized the importance of the Saudi Arabian Ministry of Sports and relevant committees making increased efforts to improve participation in sports among people with disabilities by providing better facilities and the necessary equipment and supplies. In addition, participants called for female Saudi coaches to be given opportunities to obtain comprehensive sports qualifications and extensive training similar to those available to male sports coaches. Furthermore, one participant highlighted *‘It is a necessity to have female psychologists and social workers available to assist athletes in coping with the psychological and social challenges that may negatively affect their athletic performance.’*

## 4 Discussion

The primary aim of this paper is to investigate the involvement and achievements of women with physical impairments in sports in Saudi Arabia. It aims to explore their participation, challenges, and cultural influences that shape their involvement. The paper examines the impact of societal expectations on their engagement in sports while also discussing the future of Para sports and empowerment for these women.

### 4.1 Exploring participation in Para sports

The research on sport socialization focuses on understanding how individuals become involved in the world of sports and how their beliefs and values become intertwined with it. Sport socialisation involves the adoption of attitudes, values, knowledge, and behaviors associated with sports participation (Svanelöv et al., 2020). Differentiating between being introduced to sports and learning values through sports provides a deeper understanding of why people engage in sports. This socialisation process is influenced by a combination of external factors such as location, culture, and available sporting opportunities, as well as internal factors including self-perception, life stage, learning processes, perception, motivation, attitude, and physical traits (Mullin et al., 2000; Ruddell and Shinew, 2006; Seal, 2012; Sales and Misener, 2021; Alhumaid et al., 2022; Chen et al., 2024).

This study explores the process of including women with physical impairments into Para sports in Saudi Arabia. In this study, participants recounted their introduction to Para sports, highlighting the role of societal unawareness. Their involvement stemmed from various factors such as friends’ influence, invitations to social events to join sports clubs, and information shared on social media platforms. Ruddell and Shinew (2006) identified several key factors in the socialisation of individuals with disabilities into sports, including family, school, peers, and the community. However, additional agents such as therapists, peers with disabilities, or coaches of the Para sport may also play crucial roles in introducing them to sports. Given the limited awareness about Para sports movements in Saudi Arabia, those in direct contact with individuals with disabilities, such as peers or professionals in the field, become increasingly influential in shaping their engagement in sports activities. While these factors initially prompt women with physical impairments to participate in sports, studies have shown that implementing a more structured approach is crucial to attracting and recruiting a greater number of potential women athletes with physical impairments across the country (DePauw, 2000; Ruddell and Shinew, 2006; Kirk et al., 2021).

This study also documented the process of socialisation through sports. Participants describe how engagement in Para sports had positive outcomes in their lives, such as reshaping their self-perception, bolstering confidence, cultivating a more positive outlook on life, and expanding their social connections. Previous studies have also noted that engaging in physical activity and sports contributed to psychological advantages for individuals with disabilities, enhancing their self-esteem, autonomy, goal attainment, and personal growth (García and López., 2012; Muñoz et al., 2017; Diaz et al., 2019; Aitchison et al., 2022). Involvement in Para sports enabled these women to focus on their identity, abilities, and accomplishments (Svanelöv et al., 2020; Weiller-Abels et al., 2021; Wickenden, 2023). Additionally, participants expressed their ability to inspire and motivate more women with physical impairments to participate in Para sports. Participants also mentioned how their health improved through sport, including enhanced physical fitness, reduced pain, and improved wheelchair maneuverability and mobility (Aitchison et al., 2022; Ballas et al., 2022; Ascondo et al., 2023; Zabala-Dominguez et al., 2023).

### 4.2 Challenges to participation in Para sports

The Middle East nations is working toward achieving a higher level of gender equality in the region, demonstrating a national commitment to women’s empowerment in both the public and private sectors with the advancement of policies for gender equality (Gharaibeh and Remaih, 2022). While positive advancements have been made, numerous challenges persist, impacting the involvement of women with disabilities in sports in Saudi Arabia. Understanding these barriers is crucial in fostering an inclusive and empowering environment for women with disabilities in sports.

Studies have shown that stereotypes and stigmas regarding gender and disability can lead to limited opportunities and expectations for women in sports (Coleman et al., 2015; Moodley and Graham, 2015; Weiller-Abels et al., 2021; Richard et al., 2023). In this study, participants highlighted the frequent challenges they face due to socio-cultural barriers deeply rooted in societal perceptions of individuals with disabilities, particularly women with physical impairments. As a result, the cultural and religious emphasis on doing good deeds, caring and looking after the welfare of certain groups in the community, such as orphans, the elderly, and people with disabilities has positioned these individuals as recipients of welfare outreach. For women with physical impairment, this perception clouded the opportunity of being viewed as potential investments for their development in Para sports. The results of this study align with Nagata’s (2008) assertion that the cultural perspective prevalent in the Middle East, which views efforts related to disabilities as a welfare obligation, may not be conducive to the sustainable development of Para sports. Women with physical impairment in Saudi Arabia are also faced with cultural boundaries, such as the freedom to engage in physical activities (Alhumaid et al., 2022). In addition to this, prevailing misconceptions about the capabilities of women athletes with physical impairment can hinder their access to sporting activities which include the lack of female coaches and personnel in Para sports.

Participation in Para sports can provide alternative experiences and increase visibility for women with disabilities, thereby creating greater awareness of the importance of Para sports for women athletes with physical impairments. However, participants in this study highlighted the limited funding and a lack of sponsorship opportunities often discourage them from engaging in sports, primarily due to the high costs associated with adaptive and specialized training equipment. These challenges were also highlighted in a systematic review by Olasagasti-Ibargoien et al. (2023), which examined barriers to physical activity for women with physical impairments. The study revealed that women with physical impairments encounter numerous and complex challenges. These include inadequate accessibility or adaptations in sports centers, transportation limitations to sports facilities, insufficient communication among professionals, and subpar organizational management. Furthermore, the limited space and equipment that fail to accommodate mobility restrictions hinder their ability to fully utilize exercise equipment and available space. Addressing these multifaceted barriers is crucial for creating an inclusive and supportive environment that encourages women with disabilities to actively participate in sports and physical activities.

### 4.3 Improving participation of women with physical impairments in Para sports

Participation in sports has long been recognized as a powerful tool for empowerment, confidence-building, and social inclusion (Ashton-Shaeffe et al., 2001; Pensgaard and Sorensen, 2002; Nzeyimana, 2019). However, for women with physical impairments, engaging in sports and athletic activities often comes with challenges. There is a growing recognition of the importance of inclusivity in sports, particularly in creating opportunities for women with disabilities to participate and excel in Para sports (Ashton-Shaeffe et al., 2001; Ascondo et al., 2023). Improving participation for these women in Para sports is not just about creating avenues for physical activity and sport; it is about fostering inclusivity, breaking barriers, and empowering individuals.

One of the challenges reported by the participants in this study to actively engage in sports is the lack of accessibility to suitable sporting facilities, adaptive equipment, and specialized coaching for women athletes with physical impairment. This finding were also reported in previous studies (DePauw, 2000; Nzeyimana, 2019; Sales and Misener, 2021; Olasagasti-Ibargoien et al., 2023). Most of the participants in this study commented that many sports facilities are not adequately equipped to accommodate them for training. Addressing this issue requires a concerted effort to create universally accessible environments that cater to the specific needs of women with physical impairments. This includes not only physical accessibility but also the provision of adaptive equipment and knowledgeable coaches who understand the nuances of training women athletes with physical impairment. Empowering women with physical impairments in Para sports goes beyond merely providing opportunities; it involves creating an inclusive ecosystem that celebrates diversity, recognizes individual capabilities, and promotes a culture of equality (Nagata, 2008; Chen et al., 2024). This study supports the study by Olenik et al. (1995) that identified the underrepresentation of women in Para sports, both as athletes and in administrative roles, remains a significant barrier to raising awareness about the unique challenges faced by women athletes with physcal impairments.

Societal perceptions and stereotypes about women with disabilities often act as barriers, limiting their participation in sports. These stereotypes can result in a lack of encouragement, support, media coverage, and opportunities for women with disabilities to engage in sports activities (Hamdy et al., 2011; Parsons et al., 2017; Canton et al., 2023). In this study, participants noted the absence of women with disability as role models, and that few of the participants are inspired and motivated to be role models for society through their performance in Para sport. According to Ballas et al. (2022), empowering these women requires challenging societal norms, advocating for inclusivity, and creating awareness about the capabilities and potential of individuals with disabilities in sports. Fostering a supportive and inclusive community plays a pivotal role in encouraging women with physical impairments to participate in sports. Creating networks, support groups, and mentorship programs can provide a sense of belonging, encouragement, and motivation. These platforms offer emotional support and allow sharing of experiences, strategies, and successes, inspiring others to pursue their athletic aspirations.

In addition to the physical and societal challenges, participants in this study also mentioned financial constraints as the significant barriers to participation in Para sports for women with disabilities. The cost of specialized equipment, training, and participation in sporting events can be excessively high. To encourage greater participation, it is essential to develop sustainable financial support mechanisms, scholarships, and sponsorships tailored specifically for women with physical impairments, ensuring that financial limitations do not discourage their engagement in sports.

To truly support these athletes, governing bodies within the Para sports movement must be sensitive to financial, time, and cultural constraints that specifically affect women with disabilities, who often lack institutional or interpersonal support (Alhumaid et al., 2022). Consequently, failing to sustain the involvement of today’s women athletes with disabilities will inevitably lead to a decline in participation in the future. Empowering these athletes to advocate for Para sports is challenging without opportunities for them to come together, exchange experiences, and validate each other’s journeys (Bundon and Clarke, 2015; Silva and Howe, 2018). It’s crucial to create accessible channels for women athletes to communicate their concerns to decision-makers, fostering a unified political front representing all women athletes, irrespective of their disability or sports affiliation (Powis, 2018).

Key components of organizational capacity, deemed crucial, encompass financial resources, personnel, infrastructure, operational procedures, interpersonal connections, networks, and strategic planning and advancement (Breuer and Wicker, 2014; Misener and Darcy, 2014). To encourage more participation of women with physical impairment in Para sport, requires collaborative efforts from governments, sports organizations, communities, and individuals to break down barriers and create an environment where women with disabilities can thrive in sports. Improving participation for women with physical impairments in sports in Saudi Arabia calls for a multi-faceted approach. It necessitates accessible infrastructure, awareness campaigns to challenge stereotypes, a supportive community, and financial assistance (Bundon and Clarke, 2015; Powis, 2018; Alhumaid et al., 2022). In addition, special attention should be placed on providing a women-friendly environment, such as designated women-only facilities and training arrangements. By actively addressing these challenges and promoting inclusivity, we can create a more equitable and empowering environment where women with disabilities can fully embrace the transformative power of sports, fostering their physical well-being, confidence, and sense of belonging in society (DePauw, 2000; Chen et al., 2024).

Silva and Howe (2018) commented that collectively, the Para sport community should foster a culture of multicultural exchange, welcoming diverse perspectives and innovative thinking. This approach should aim to empower individuals socially, acknowledging and celebrating the wide spectrum of human experiences. Given the tendency to overlook athletes with high support needs and exclude them from mainstream Paralympic events, it is imperative to focus on expanding opportunities and promoting inclusivity for these athletes. Additionally, heightened awareness is needed regarding the compounded challenges faced by women with physical impairments (Silva and Howe, 2018).

Participation in Para sports is not only a testament to human resilience but also a reflection of intrinsic and extrinsic motivational factors that drive individuals with disabilities to engage in athletic pursuits. These motivators play a pivotal role in inspiring and empowering individuals to overcome challenges and actively participate in sports, fostering personal growth, and contributing to the wider community.

## 5 Study limitations

It is important to acknowledge the limitations of this study’s design when interpreting its findings. First, the reliance on participants’ self-reported answers to posed questions introduces the possibility of “social desirability bias.” This bias occurs when participants feel compelled to conform to societal expectations or endorse decisions that support Para sports for women with disabilities in Saudi Arabia. However, the study implemented measures to mitigate this bias, including confidentiality assurances and a purely scientific objective. Second, the study’s small sample size focused exclusively on individuals with physical impairments. While this choice was based on the prevalence of physical impairments among Saudi women who engage in physical activities and sports, it is not possible to generalize these findings to all individuals with physical impairments. In other words, the current study’s findings may not represent the overall reality of sports for people with disabilities, including those with physical impairments, in Saudi Arabia. Nevertheless, the study’s results have provided valuable insights and recommendations that can significantly enhance the effectiveness and success of Saudi women with disabilities in engaging in physical and sporting activities. To gain a more comprehensive understanding of the experiences and challenges faced by individuals with disabilities in sports, future research could assess any changes or improvements over time, providing a more nuanced understanding of the dynamics of sports participation among Saudi women with physical impairments. Finally, the study has one last limitation, which is the distinction between the participants involved in individual Para sports and those engaged in team Para sports. Therefore, future research should consider this aspect, as the circumstances and nature of participation in individual sports and team sports differ.

## 6 Conclusion

Participation in sports is a powerful avenue for empowerment and social inclusion. However, women with physical impairments face unique challenges in accessing sports. Women with physical impairments in Saudi Arabia encounter challenges due to inadequate facilities, equipment, and coaching. Creating universally accessible environments with specific equipment and knowledgeable coaches is pivotal in addressing this issue. Additionally, creating a sporting environment that encourages women to participate in sports is also important, such as women only training schedules, and more certified women coaches or team managers for women athletes with a physical impairment. Financial constraints also pose significant obstacles in Para sport. Developing and providing financial support mechanisms ensures that women with disabilities can overcome these barriers and engage fully in sports. Participation in Para sports is not only a testament to human resilience but also a reflection of intrinsic and extrinsic motivational factors that dritve individuals with disabilities to engage in sports. These motivators play a pivotal role in inspiring and empowering individuals to overcome challenges and actively participate in sports, fostering personal growth, and contributing to the wider community. Empowerment in Para sports necessitates an inclusive ecosystem that celebrates diversity and equality. Collaborative efforts from governments, sports bodies, communities, and individuals are essential in creating an environment where women with disabilities can thrive in sports.

## Data availability statement

The raw data supporting the conclusions of this article will be made available by the authors, without undue reservation.

## Ethics statement

The studies involving humans were approved by Research Ethics Committee at King Faisal University in Saudi Arabia (Protocol code: KFU-REC-2023-JUN-ETHICS1091). The studies were conducted in accordance with the local legislation and institutional requirements. The participants provided their written informed consent to participate in this study.

## Author contributions

MMA: Conceptualization, Data curation, Formal analysis, Funding acquisition, Investigation, Methodology, Project administration, Resources, Supervision, Validation, Visualization, Writing – original draft, Writing – review & editing. YA: Conceptualization, Data curation, Investigation, Methodology, Resources, Supervision, Validation, Visualization, Writing – original draft, Writing – review & editing. MS: Formal analysis, Investigation, Methodology, Resources, Validation, Visualization, Writing – review & editing. MAA: Data curation, Investigation, Resources, Validation, Visualization, Writing – review & editing. SK: Conceptualization, Investigation, Resources, Supervision, Validation, Visualization, Writing – original draft, Writing – review & editing.
